# Heterotopic pancreas in the omphalomesenteric duct remnant in a 9-month-old girl: a case report and literature review

**DOI:** 10.1186/s13000-017-0643-2

**Published:** 2017-07-05

**Authors:** Zitong Zhao, Chiang Khi Sim, Sangeeta Mantoo

**Affiliations:** 1 Department of Anatomical Pathology, Division of Pathology, Singapore General Hospital, Singapore, Singapore; 2 Pediatric Surgery, Mount Elizabeth Medical Centre, Singapore, Singapore

**Keywords:** Heterotopic pancreas, Omphalomesenteric duct remnant

## Abstract

**Background:**

Heterotopic pancreas most commonly occurs in the upper gastrointestinal tract of adults, usually as an incidental finding. It seldom occurs at the umbilicus, and even rarely in the pediatric age group.

**Case presentation:**

Here we present a case of heterotopic pancreatic tissue in the omphalomesenteric duct remnant of a 9-month-old baby girl. She presented with redness at the base of the umbilicus associated with occasional mild wetness. A urachal fistula was suspected by ultrasound. Histology from subsequent resection revealed fibrous tissue with heterotopic pancreatic tissue and accompanying small bowel mucosa. The patient’s umbilical redness resolved after the surgery.

**Conclusions:**

Upon literature search, we found only 17 other cases of heterotopic pancreas reported in the umbilicus. They described a high male to female ratio, frequent association with omphalomesenteric duct remnant and presentation of umbilical discharge. The Heinrich system is frequently used to classify heterotopic pancreas into 3 types, based on the presence of acini, islets and ducts. Several mechanisms have been proposed on the pathogenesis of heterotopic pancreas, including misplacement, metaplasia and totipotent cell theories. Heterotopic pancreas can manifest clinically with diseases of the pancreas, including malignant transformation, reported as high as 12.7% in a series. Awareness of this finding in the biopsy aids the suitable treatment decisions for the patient.

## Background

Umbilical discharge is a common pediatric problem. An underlying congenital anomaly should always be considered. Heterotopic pancreas is the second most common congenital anomaly of the pancreas, most commonly occurring in the upper gastrointestinal tract of adults. It seldom occurs at the umbilicus, and even rarely in the pediatric age group. Here we present a 9-month-old baby girl with heterotopic pancreatic tissue in the omphalomesenteric duct (OMD) remnant. Literature review of other 17 cases of heterotopic pancreas reported at the umbilicus has been performed [1–12].

## Case presentation

### Clinical presentation

**Fig. 1:**
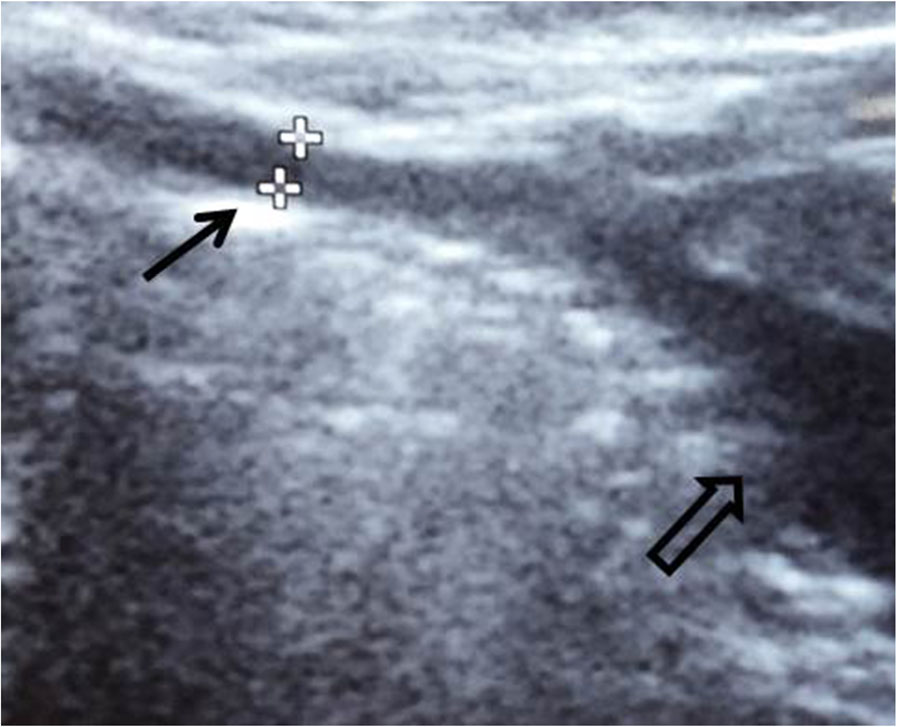
Ultrasound findings. There was a tubular structure (*black arrow*) traced from umbilicus with possible connection to the urinary bladder (*hollow arrow*)

A 9-month-old baby girl presented with redness at the base of the umbilicus since her umbilical cord fell off, without accompanying discharge or smell. However, there was mild wetness in the umbilicus when she cried. The mother experienced an uneventful pregnancy and delivery. No congenital anomalies were discovered. There was no other medical history or family history of medical issues. Ultrasound revealed a tubular structure at the umbilicus with possible connection to the bladder (Fig. 1). It was clinically suspicious for a urachal fistula. The patient underwent surgery. Intraoperatively, a fibrous cord was traced from the umbilicus, but no obvious nodules were observed. There was no communication of the fibrous cord with the urinary bladder, bowel or any other intra-abdominal structure. This fibrous cord was therefore excised.

### Gross and histological findings

**Fig. 2:**
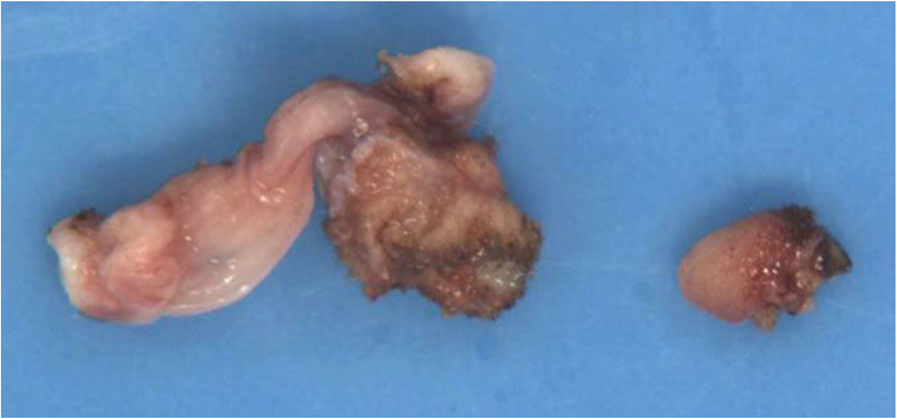
Gross appearance. There was a tubular structure and a separate piece of mucosa

**Fig. 3:**
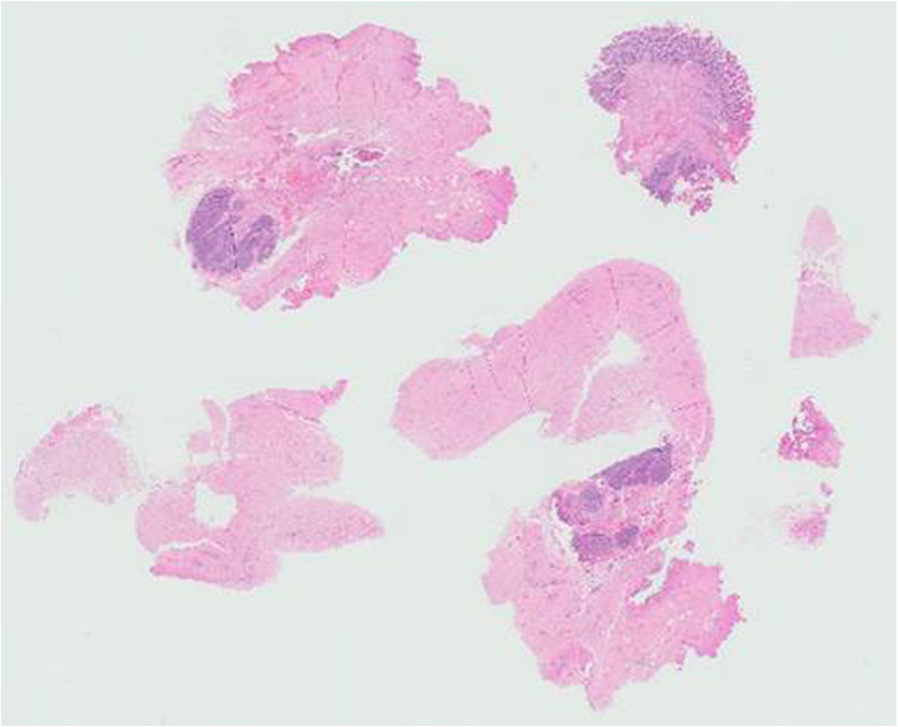
Whole mount view. There were multiple fragments of fibrotic tissue and a piece of small bowel mucosa (original magnification ×2.5)

**Fig. 4:**
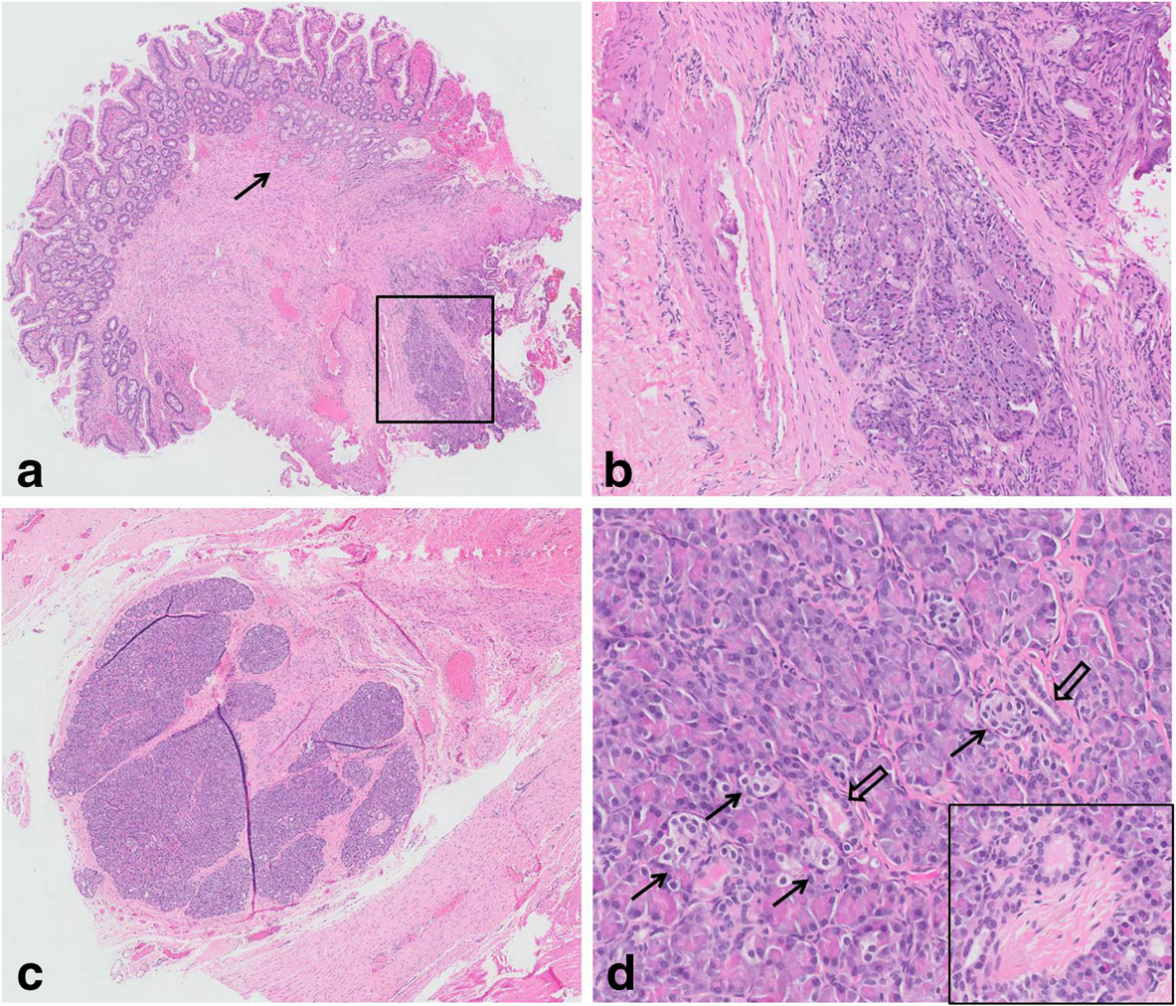
Haematoxylin and Eosin (H&E) micrographs. The small bowel mucosa showed generally intact villous architecute, with Brunner’s glands observed in the laminia propria (*black arrow*). Smooth muscle bundles were haphazardly arranged in the underlying stroma, while no well-developed muscularis propria were seen (**a**, original magnification ×20). At the cauterized edge, there were lobules of pancreatic tissue, comprising benign acini and islets of Langerhans (**b**, ×100). A larger focus of pancreatic tissue was present in the fibrotic tissue (**c**, ×20). All three components were observed, including acini, islets of Langerhans (*black arrow*) and ducts (*hollow arrow*) (**d**, ×200). An intralobular nerve bundle was focally seen, adjacent to small ducts (inset, ×200)

**Fig. 5:**
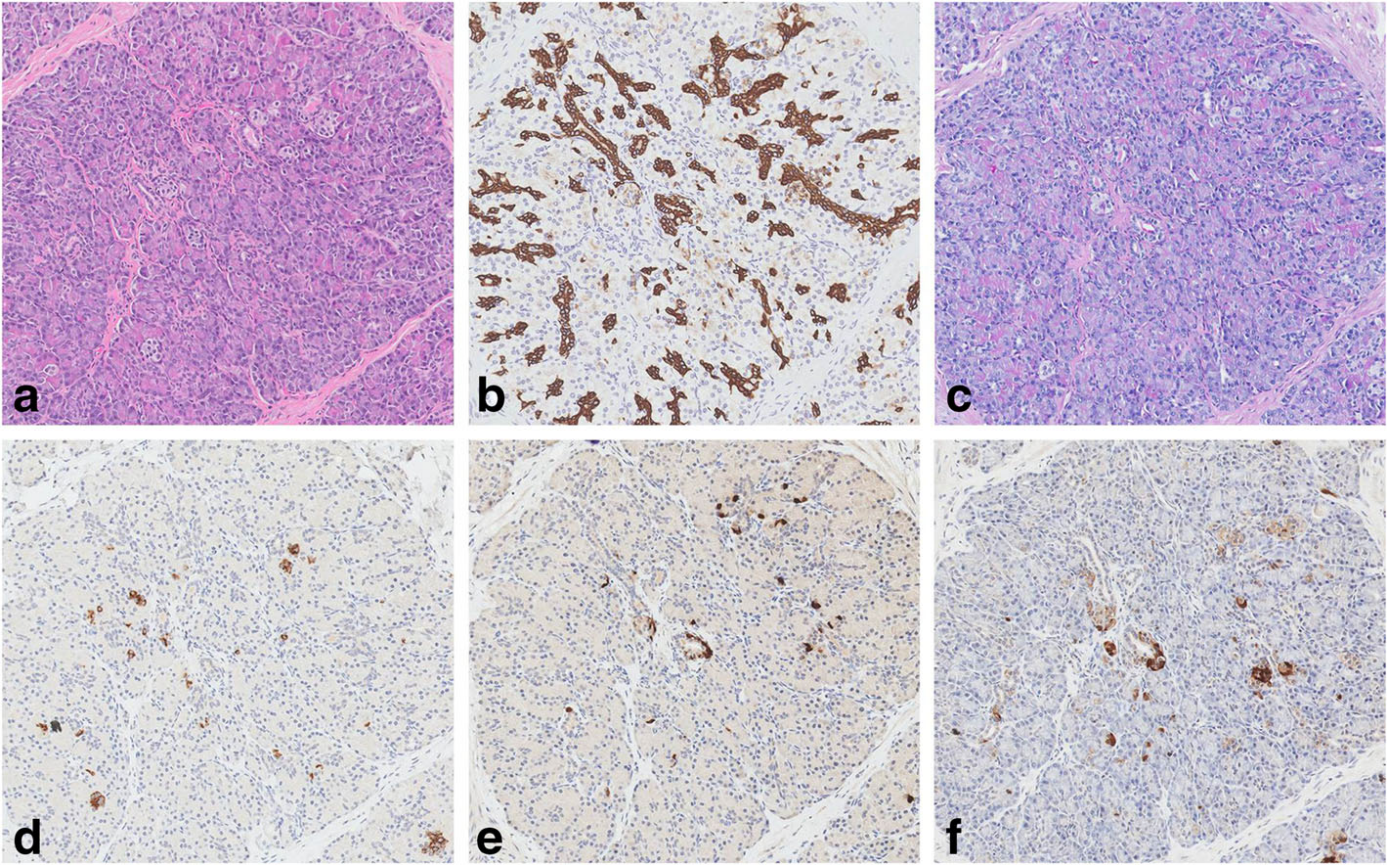
Heterotopic pancreatic components. The haematoxylin and eosin micrograph showed exocrine and endocrine structures of pancreas (**a**, original magnification ×10). Ducts and pancreatic acini were highlighted by pan-cytokeratin marker AE1/3 immunostaining (**b**, ×10) and Periodic acid–Schiff–diastase special stain (**c**, ×10) respectively. The distribution of insulin-, glucagon-, and somatostatin-producing neuroendocrine cells scattered in the islets were demonstrated with respective immunohistochemistry (**d** to **f**, ×10)

Two separate pieces of unoriented tissue were received for histology, measuring 2 × 1 × 0.3 cm and 0.4 × 0.4 × 0.3 cm respectively. The larger piece revealed a possible sinus-like tract measuring 0.5 cm in length and 0.2 cm in diameter (Fig. 2). Haematoxylin and eosin sections showed fragments of dense fibrotic tissue with lobules of heterotopic pancreatic tissue comprising acinar elements, ducts and endocrine type cells (Figs. 3 and 4). The different components were demonstrated with immunohistochemical and special stains (Fig. 5). The smaller fragment also showed a covering small bowel mucosa with a deeply located tiny portion of pancreatic tissue close to the base of resection (Fig. 4a). No malignancy was identified.

## Discussion

Umbilical discharge is common in children and usually due to infection. An underlying congenital anomaly should be investigated, such as umbilical hernia, urachal remnant and OMD remnant. OMD remnant, with a prevalence of only 2%, varies from patent OMD, to cysts, fibrous cords connecting the umbilicus to the distal ileum, granulation tissue at the umbilicus, umbilical hernias, and diverticulum of Meckel [13]. OMD remnant causes umbilical discharge generally through patency between the gut and umbilicus, rarely due to the presence of heterotopic pancreas.

First described in 1729 by Jean-Schultz, heterotopic or ectopic pancreas is defined as the presence of an abnormally located focus of normally developed pancreatic tissue outside the boundaries of the orthotopic pancreas, without anatomical or vascular connections. It is considered as the second most common congenital anomaly of the pancreas, after divisum. The prevalence has been reported to be 0.55 to 13.7% at autopsy and 0.2% at laparotomy [14]. It is most often discovered in fifth and sixth decades of life, and infrequently reported in the pediatric age group [11]. Seventy to 90% of heterotopic pancreas occurs in the upper gastrointestinal tract, while it can also be found anywhere [15]. It usually presents as an asymptomatic incidental finding, but it can cause symptoms as well depending on the size, location and pathological changes.

Umbilicus is an uncommonly reported location for heterotopic pancreas. Only 18 cases were found at the umbilicus, including ours (Table 1). The age ranges from newborn to 60 years old. Fifteen of them were below 2 years old, one adolescent, one young adult and only one elderly. Fourteen were male and 3 were female, one not indicated in the report. Most of them presented with umbilical discharge. Five were described to have coexisting small intestinal and/or gastric mucosa, which suggested the presence of heterotopic pancreas in the OMD remnant.

**Table 1:** Table 1. Summary of cases of heterotopic pancreas at the umbilicus

No.	Age/Sex	Umbilical discharge	Heinrich type	Other pathological findings	Reference
1	12 years/F	N/A	N/A	N/A	Wright (1900), cited by Harris et al. [1]
2	22 years/M	Y	N/A	N/A	Trimingham (1943), cited by Harris et al. [1]
3	Newborn	N	I	N/A	Harris et al. (1963) [1]
4	6 months/M	N/A	N/A	N/A	Steck and Helwig (1964), cited by Avolio et al. [4]
5	13 months/M	Y	N/A	N/A	Caberwal et al. (1977) [2]
6	60 years/M	N	II	Small intestinal mucosa	Kondoh et al. (1994) [3]
7	8 months/M	Y	N/A	N/A	Avolio et al. (1998) [4]
8	15 months/M	Y	N/A	N/A	Avolio et al. (1998) [4]
9	6 months/M	Y	N/A	N/A	Perez-Martinez et al. (1999) [5]
10	3 months/M	Y	I	N/A	Tan et al. (2000) [6]
11	7 weeks/M	Y	I	N/A	Tan et al. (2000) [6]
12	2 years/M	N/A	N/A	N/A	Tillig et al. (2004) [7]
13	8 days/M	Y	I	Acute haemorrhage	Lee et al. (2005) [8]
14	18 months/M	Y	N/A	Gastric mucosa	Silva et al. (2010) [9]
15	2 years/M	N	N/A	N/A	Abdelgabar et al. (2013) [10]
16	2 years/M	Y	I	Small intestinal mucosa and gastric mucosa	Sharma et al. (2013) [11]
17	3 months/F	Y	I	Small intestinal mucosa	Park et al. (2014) [12]
18	3 months/F	Y	I	Small intestinal mucosa	Present case

*Abbreviations*: *N/A* not available, *Y* yes, *N* no

The Heinrich system is frequently used to classify heterotopic pancreas into 3 types. Type 1 contains acini, islets and ducts. Type 2 contains acini and ducts only, but no islets. Type 3 contains ducts alone [16]. In the described heterotopic pancreas at the umbilicus, type 1 was most commonly encountered.

Several mechanisms have been proposed for the pathogenesis of heterotopic pancreas, including the misplacement of embryonic tissue developing into pancreatic tissue, the metaplasia of endodermal tissue that migrates to the submucosa during embryogenesis into pancreatic tissue, and the differentiation of totipotent endodermal cells lining the gut or OMD into pancreatic tissue [12].

Heterotopic pancreas can manifest clinically with diseases of the pancreas, such as pancreatitis, pancreatic cyst, neuroendrocrine tumour and pancreatic carcinoma [17]. The incidence of malignant transformation is reported as high as 12.7% in a Japanese series [18]. Therefore, follow up is suggested if incompletely excised, although heterotopic pancreas itself is a benign condition. In this case, heterotopic pancreatic tissue was present at the cauterized edge of the specimen, which raised the possibility of residual pancreatic tissue left behind. Till now, on the follow-up of the patient, her umbilical redness resolved. Limited local excision appeared to be a safe and adequate procedure in the current setting.

## Conclusions

In conclusion, heterotopic pancreas at the umbilicus is an uncommon condition, predominantly occurring in infants despite a wide range of ages. It demonstrates a high male to female ratio, frequent association with omphalomesenteric duct remnant and presentation of umbilical discharge. Radiology may not be helpful. Diagnosis is usually straight forward on the histologic evaluation of resection specimen, complemented with immunohistochemistry. Awareness of this finding in biopsy aids in the suitable treatment decisions for the patient.

## Abbreviation

OMD: omphalomesenteric duct
